# Crowdfunding the *Azolla* fern genome project: a grassroots approach

**DOI:** 10.1186/2047-217X-3-16

**Published:** 2014-09-26

**Authors:** Fay-Wei Li, Kathleen M Pryer

**Affiliations:** 1 Department of Biology, Duke University, Durham, North Carolina 27708, USA

**Keywords:** *Azolla*, Cyanobacteria, Fern, Genome sequencing, Nitrogen fixation, *Nostoc*, Op-ed, Social media, Symbiosis, Crowdfunding

## Abstract

Much of science progresses within the tight boundaries of what is often seen as a “black box”. Though familiar to funding agencies, researchers and the academic journals they publish in, it is an entity that outsiders rarely get to peek into. Crowdfunding is a novel means that allows the public to participate in, as well as to support and witness advancements in science. Here we describe our recent crowdfunding efforts to sequence the *Azolla* genome, a little fern with massive green potential. Crowdfunding is a worthy platform not only for obtaining seed money for exploratory research, but also for engaging directly with the general public as a rewarding form of outreach.

## Background

*Fern genomics: the final frontier. These are the voyages of Team Azolla. Its three-year mission: to explore strange new genome space, to seek out new genes and new functions, to boldly go where no fern has been sequenced before.* – Modified from Captain James T. Kirk, Star Trek.

Most of the 470 million year history of plants on land belongs to bryophytes, lycophytes, ferns, and gymnosperms, which eventually yielded to the infamous dominance of flowering plants 90 million years ago. Ferns, the third largest of these five radiations—and the sister group to all seed plants—are the only lineage for which a reference genome has not yet been sequenced. That doesn’t sound right, does it? Without a fern genome, one cannot expect to fully comprehend the processes that govern the evolution of plant genes and genomes, and the patterns underlying major evolutionary transitions in land plants will remain elusive [1].

**Figure 1:**
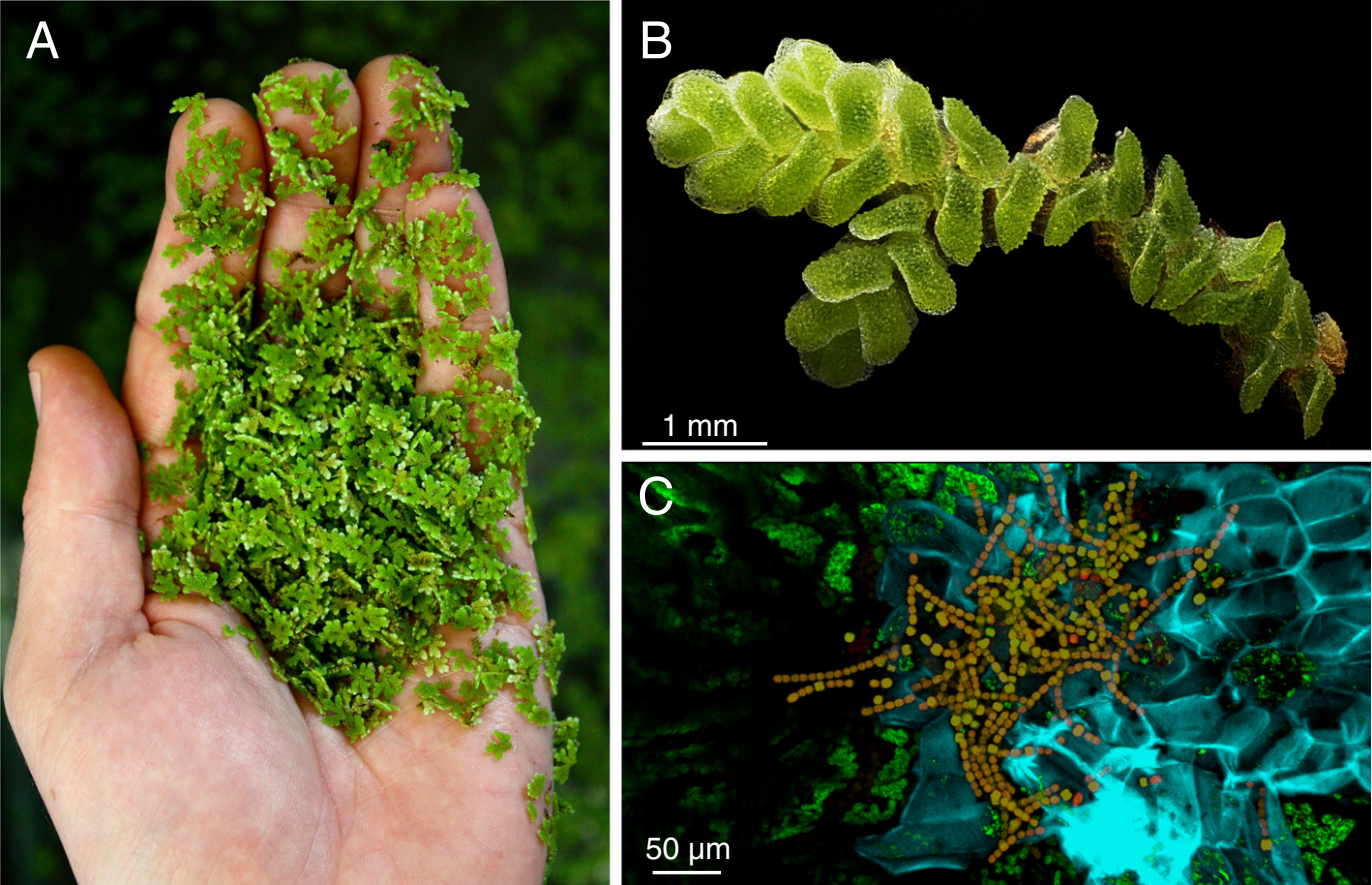
***Azolla *is a small aquatic fern with an obligate bacterial endosymbiosis. A**: hundreds of *Azolla* individuals can sit in the palm of your hand. **B**: close-up of an *Azolla* plant. Photo credit: Mark Smith and Dan Saftner of Macroscopic Solutions. **C**: *Nostoc,* symbiotic cyanobacteria (reddish brown filaments) living inside *Azolla* leaf cavities. Photo credit: Jerald Pinson.

Important guidelines for selecting past candidates for genome sequencing have included their economic relevance to humans, their potential to answer important biological questions, and a small genome size. *Azolla*, a small genus of floating aquatic ferns (Figure 1), passes all three criteria with flying colors. First, Asian farmers have been using *Azolla* for thousands of years as a companion crop for rice—the world’s most important food staple [2]. *Azolla* is a natural nitrogen biofertilizer, a green manure, that bolsters rice productivity when these two plants are grown together [3,4]. Furthermore, *Azolla* has been shown to be capable of hyperaccumulating a great variety of heavy metal pollutants, as well as decontaminating superfluous ammonium and phosphorus in wastewater [5,6]. Second, fossil data from the Arctic Ocean demonstrate that around 50 million years ago, there was an enormous *Azolla* bloom, spanning nearly a one million-year-interval, that sequestered over 10^12^ tons of carbon and helped to shift the Early Eocene greenhouse world towards our present icehouse climate [7,8]. Could *Azolla* help to do this again? Lastly, the 750 Mb genome of *Azolla* is tiny compared with other fern genomes, which are typically >10Gb [9], making it the perfect first genome to be sequenced in ferns. We believe that *Azolla* genomic studies will not only improve our understanding of plant evolution, but will also have broad implications for sustainable agriculture, phytoremediation and biofuel production.

*Azolla* is more than just a fern; it is a superorganism. Within specialized cavities enclosed by each of its tiny leaves, *Azolla* harbors an obligate symbiont, the nitrogen-fixing cyanobacterium *Nostoc (Anabaena) azollae* (Figure 1C). The *Azolla—Nostoc* interaction is unique among all known plant—bacterial symbioses in that the association is maintained perpetually, even through sexual reproduction, so that the *Nostoc* cyanobiont is vertically transmitted to subsequent generations. In fact, *N. azollae* appears to have entirely lost its autonomy, as it cannot be cultured independently, that is when isolated from its host plant. A recent study revealed that *N. azollae* has undergone genomic downsizing, suggesting it might be in the early stages of evolving into a plant organelle devoted to nitrogen fixation [10]. However, this somewhat well characterized *Azolla—Nostoc* symbiosis is just the tip of the *Azolla* microbiome iceberg. In addition to *Nostoc*, an entire community of other bactobionts exists within *Azolla*’s specialized leaf cavities, including *Arthrobacter, Agrobacterium* and *Corynebacterium*. Interestingly, some of these non-*Nostoc* symbionts may also have nitrogen-fixing capabilities [11], suggesting that they may contribute to *Azolla*’s capacity for nitrogen fixation. This microbial community is very poorly characterized; the diversity and identity of these other bacteria, the degree of their interaction with the host, their mode of inheritance and patterns of co-evolution have not yet been investigated.

Our basic frustration of working with a plant lineage that the genomics world is blind to—one that seemed destined to be forever without appropriate genetic resources—drove us to propose the *Azolla* Genome Project. Here we aim to (1) generate a reference genome for *Azolla*, as well as for the metagenomes of its symbiotic bacteria; (2) resequence *Azolla* and its symbionts across all known *Azolla* species to study genomic co-evolution; (3) use comparative transcriptomics to infer the functionality of the symbionts and to identify candidate genes essential to this symbiosis and the nitrogen-fixation process; and (4) revisit plant genome and gene family evolution with this very first genome for the fern lineage.

Because this project repeatedly fell outside of the interest scope of major funding agencies, we decided to experiment with the “crowdfunding” route, seeking support and backing from the general public. It has been a fascinating ride, and in this commentary we share our experiences and argue that crowdfunding is a great medium for science outreach.

## Main text

### Our *Azolla* crowdfunding campaign

The ultimate goal of crowdfunding is to garner sufficient interest and enthusiasm from a large enough group of people over the Internet, so that they feel compelled to provide some measure of funding toward your project. Reaching out and convincing a total stranger more than 6,000 miles away to pitch in sounds like a daunting task, and indeed it was. We struggled and learned a lot as we went along.

For the past five years, people have been using websites, such as Kickstarter and Indiegogo, to crowdfund their creative arts, tech or gaming projects. We chose a relatively new platform, Experiment.com, because it is completely science oriented. One potential drawback is that Experiment has a much narrower audience compared to those of the large and hyper-diverse Kickstarter or Indiegogo platforms; therefore, one’s project could have less visibility to potential backers from outside of science. At Experiment, we set our campaign duration to 40 days and our funding goal to $15,000, with an objective to be able to draft an *Azolla* genome with 100X coverage [12].

Within our first naïve moments of launching the campaign, we seriously thought we could just kick off our shoes, sit back and watch the cash flow in. Well, we were completely mistaken. It was the start of a constant marathon of social media networking through email, Twitter, Facebook, and other resources we had never heard of before. Based on our experience, there are at least three major social network layers that one needs to penetrate. The first layer comprises immediate families, friends and colleagues; happily they were also among the first to donate (thanks Mom!). We asked them to tell their friends, families and colleagues so that we could extend our reach to the second layer—acquaintances. By repeating and extending this cycle, eventually our message reached the most important third layer, consisting of complete outsiders.

Into the second week of our crowdfunding campaign, we discovered the power of Reddit, an online news hub and discussion forum with enormous visitor traffic. We did a Reddit Science AMA (Ask Me Anything), where anyone could ask us any question in one afternoon about *Azolla*, ferns, plants—even career advice! We answered more than 200 questions that afternoon and got over 3,000 “upvotes”. The number of visitors to our crowdfunding website spiked during that afternoon and the following two days. To reach out and directly communicate with a broad and diverse audience, a Reddit AMA is something we highly recommend.

Obviously, not everyone surfs Reddit, or keeps up with the latest trends on social networks. We therefore also reached out using a more traditional news medium: newspapers. At the urging of Duke University’s Office of News & Communications, the junior author wrote an op-ed stressing the importance of *Azolla* research. It was immediately picked up by Canada’s highest-circulating newspaper, The Toronto Globe and Mail, as well as the Contra Costa Times of the San Francisco Bay area [13]. A fair number of our backers were from Canada, which we believe can be attributed to the op-ed in The Globe and Mail.

As this momentum began to build, we were also fortunate enough to get serious attention from the “big guys”—The Economist, USA Today and Scientific American [13]—who all reported on our efforts. Such coverage, without a doubt, gave us a tremendous push.

### Our results

Within 40 days, we had 6,191 page views on our crowdfunding website, with visitors from 65 different countries. The top-five visitor sources included referrals from Twitter, Reddit, and Facebook, indicating that social networks and Reddit indeed played important roles. We raised a total of $7,160 from 123 backers, with individual donations ranging from $1 to $1,000. We calculated that 79% of the backers were from outside of our family-friends-colleagues-acquaintances circles, and they contributed 58% of the pledges.

Halfway through the campaign, and having reached one-third of our funding goal, our journey took an unexpected turn: BGI (formerly the Beijing Genomics Institute, but now based in Shenzhen) fully backed our project. BGI did not pledge actual money; instead they offered to fulfill all our sequencing needs free of charge. Such collaboration will allow us to go well beyond a single draft genome; we plan to also use genome/metagenome resequencing and comparative transcriptomics to further elucidate the genomic basis of symbiosis and nitrogen fixation.

But what did that mean for our crowdfunding project? We could not have created the media press and excitement that caught BGI’s interest without the help of our backers. Did we need to withdraw our campaign? We contacted the founders of Experiment who told us that because our BGI backing would allow us to meet the goals of our project, that they would indicate on the website that the project was a go. After announcing the backing from BGI, we continued to raise money, in the form of a “stretch-goal”, for fueling the next phase of our research. We will use the crowdfunded pledges to build a high-resolution genome map that will greatly enhance the final assembly of the reference genome.

## Conclusion

Crowdfunding is a truly rewarding platform for engaging the public and obtaining seed money for exploratory research. It was a fantastic outreach and learning experience for us. We were forced to explain our ideas in a simple and interesting way that non-scientists would appreciate (otherwise no money!). The public endorsement and confirmation by the media that our project was of significance, and reflected what the public does care about, led to our vision being realized by BGI.

We will continue to share our latest research updates and plans with our 123 backers (affectionately known as “Team *Azolla*”), and we believe they are enjoying the ride as much as we are. With such a grassroots approach, we hope to demonstrate it is possible (not to mention important) to bring the general public closer to how the process of scientific inquiry works.

## Competing interests

The authors declare that they have no competing interests.

## Authors’ contributions

FWL and KMP wrote the article. Both authors read and approved the final manuscript.
